# Supplementary material for: External Validation of the Colon Life Nomogram for Predicting 12-Week Mortality in Dutch Metastatic Colorectal Cancer Patients Treated with Trifluridine/Tipiracil in Daily Practice
Source: Cancers (Basel). 2022 Oct 18;14(20):5094. doi: 10.3390/cancers14205094 (PMC9599794; doi:10.3390/cancers14205094)
Supplement: Supplementary file 1 [file cancers-14-05094-s001.zip › cancers-1894276-File S1.pdf]

File S1. Formulas to predict 12-week mortality risk following diagnosis of refractory metastatic colorectal cancer (Colon Life nomogram).

The specifications for the Colon Life nomogram were obtained via the authors of the original publication.

Reference: Pietrantonio *et al.* Estimating 12-Week Death Probability in Patients with Refractory Metastatic Colorectal Cancer: The Colon Life Nomogram. *Ann. Oncol.* **2017**, *28*, 555-561.

Prognostic index (PI):

PI = $-2.6025 + 0.6964*X_{\text{No primary tumour resection}}$
$+ 1.0033*X_{\text{ECOG Performance Status = 1}} + 2.0567*X_{\text{ECOG Performance Status = 2}}$
$+ f\,(\text{LDH})$
$+0.4989*X_{\text{Peritoneal metastasis}}$

Where LDH is described with a restricted cubic spline function:

$f\,(\text{LDH})$ = $0.0018*X_{\text{LDH}}-8.3612*10^{-10}*\max(X_{\text{LDH}}-165)^3+1.0159*10^{-9}*\max(X_{\text{LDH}}-271)^3$
$-1.7977*10^{-10}*\max(X_{\text{LDH}}-764)^3$

The absolute predicted 12-week mortality risk:

Risk = 1 / (1 + exp(-PI))

Example:

The 12-week predicted mortality risk for a patient who received a primary tumour resection and who has refractory metastatic colorectal cancer with a performance score of 2, LDH value of 792 and who was diagnosed with peritoneal metastases:

PI = $-2.6025 + 2.0567*X_{\text{ECOG Performance Status = 2}} + 0.0018*(792)$
$-8.3611807*10^{-10}*\max(792-165)^3 + 1.0158919*10^{-9}*\max(792-271)^3$
$-1.7977387*10^{-10}*\max(792-764)^3 + 0.4989*X_{\text{Peritoneal metastasis}}$

= $-2.6025 + 2.0567 + 0.0018*(792)$
$-8.3611807*10^{-10}*627^3 +1.0158919*10^{-9}*521^3-1.7977387*10^{-10}*28^3$
$+ 0.4989$

= 1.3163

Risk = 1 / (1 + exp(-1.3163))
= 0.7886
