# Supplementary material for: External Validation of the Colon Life Nomogram for Predicting 12-Week Mortality in Dutch Metastatic Colorectal Cancer Patients Treated with Trifluridine/Tipiracil in Daily Practice
Source: Cancers (Basel). 2022 Oct 18;14(20):5094. doi: 10.3390/cancers14205094 (PMC9599794; doi:10.3390/cancers14205094)
Supplement: Supplementary file 1 [file cancers-14-05094-s001.zip › cancers-1894276-Supplementary Figures and Tables.pdf]

Supplementary Table S1. QuPath specifications for TK1 expression analysis

**Positive cell detection**	Using the optical density sum and requested pixel size 0.5 µm.
**Nucleus parameters**	Background radius 8 µm, median filter radius 0 µm. sigma 1.5 µm, minimum area 10 μm^2 and maximum area 400 μm^2.
**Intensity parameters**	Threshold 0.07, maximum background intensity 2, split by shape.
**Cell parameters**	Cell expansion up to 7.4468 µm and include cell nucleus.
**Intensity threshold parameters**	Score compartment: cell DAB OD mean.<br><br>Threshold +1 (0.081), +2 (0.19), +3 (0.5).

The positive cell detection parameters used for the TK1 expression analysis in QuPath are specified in the table.

Supplementary Figure S1. Calibration plots of our external validation cohort alongside previously published external validation cohorts

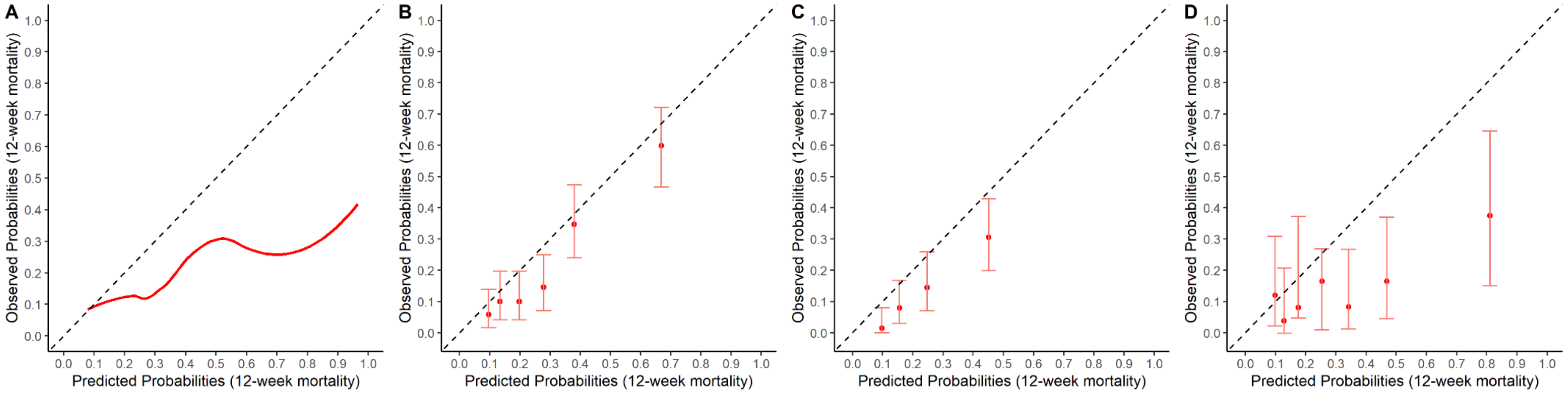

The calibration plots of predicted 12-week mortality probability versus observed 12-week mortality probability are shown for our external validation cohort (A) and as published for the Italian refractory mCRC patients from 12 institutions (external validation cohort)[5] (B) Italian compassionate use program cohort[6] (C) and the RECOURSE trial participants (D)[7]. The published calibration plots were digitized using WebPlotDigitizer version 4.4.

Supplementary Figure S2. Receiver-operator curve for the addition of QoL-SS to the Colon Life nomogram in QUALITAS patients

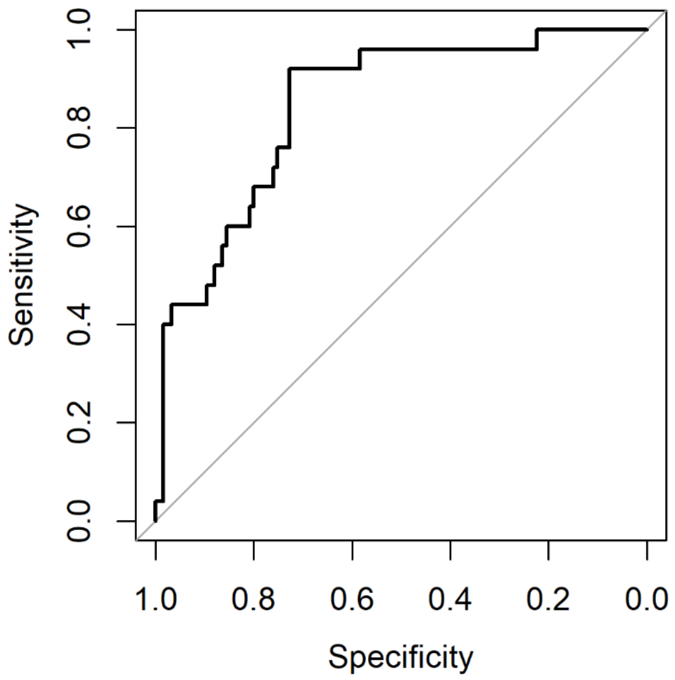

Supplementary Figure S3. Calibration plot of the Colon Life nomogram with QoL-SS predicted versus observed 12-week mortality in QUALITAS patients

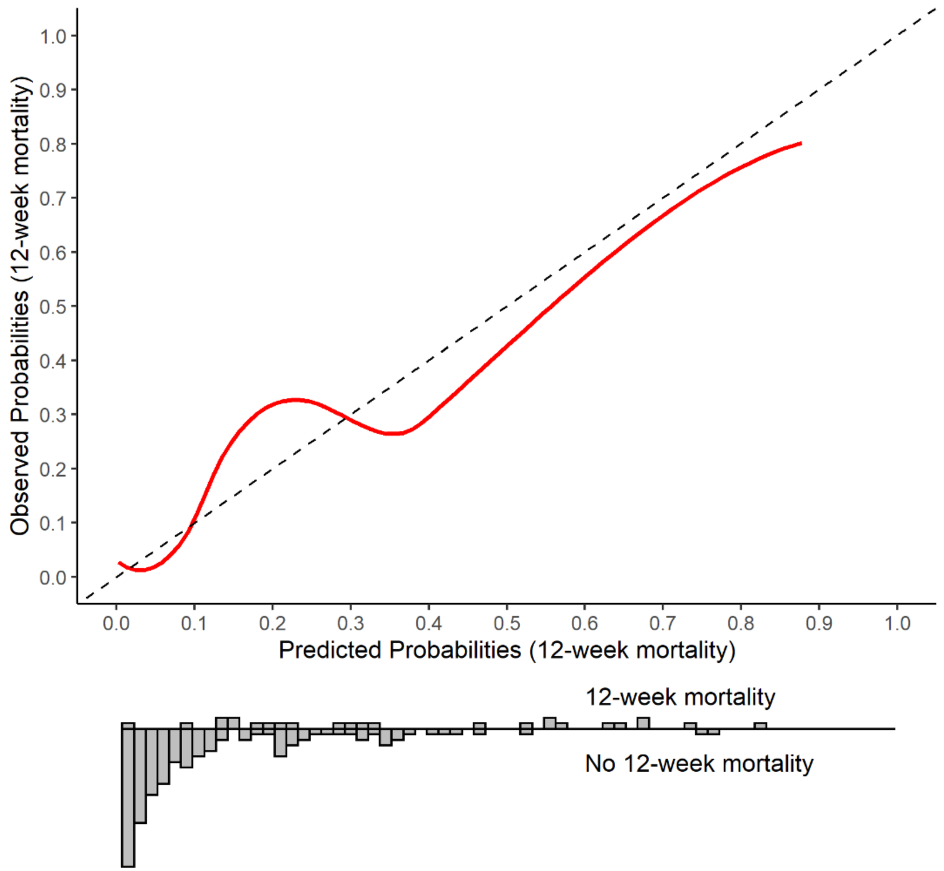

The Colon Life model with baseline QoL-SS predicted 12-week mortality versus the observed 12-week mortality is shown, with the diagonal line indicating performance of a well-calibrated model. The histogram displays the predicted probability distribution for 12-week mortality risk for patients who died within 12 weeks of initiating FTD/TPI treatment versus who survived.

Supplementary Figure S4. Flow diagram for QUALITAS patient tissue samples analyzed for TK1 expression

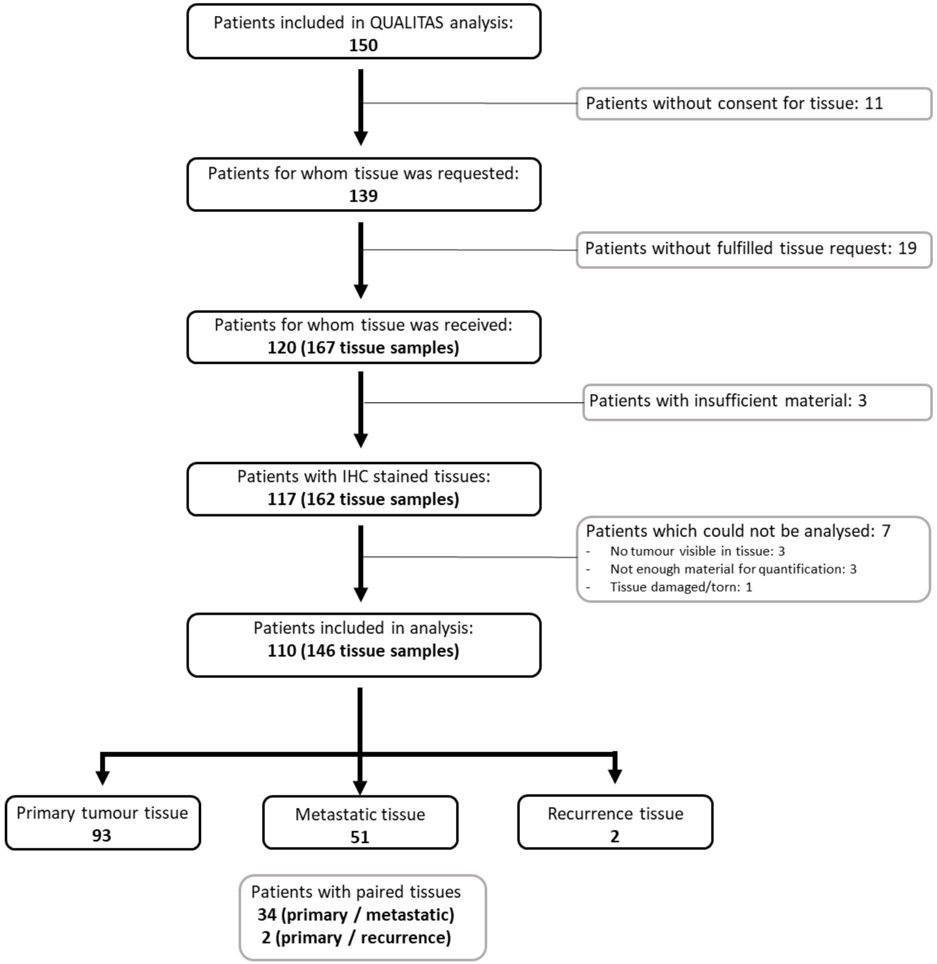

Flow diagram illustrating the QUALITAS patients for which archival tissue could be requested from PALGA, tissue received and tissue which could be analyzed for TK1 expression levels.

Supplementary Figure S5. TK1 expression levels in paired primary – metastatic – recurrence tissue samples

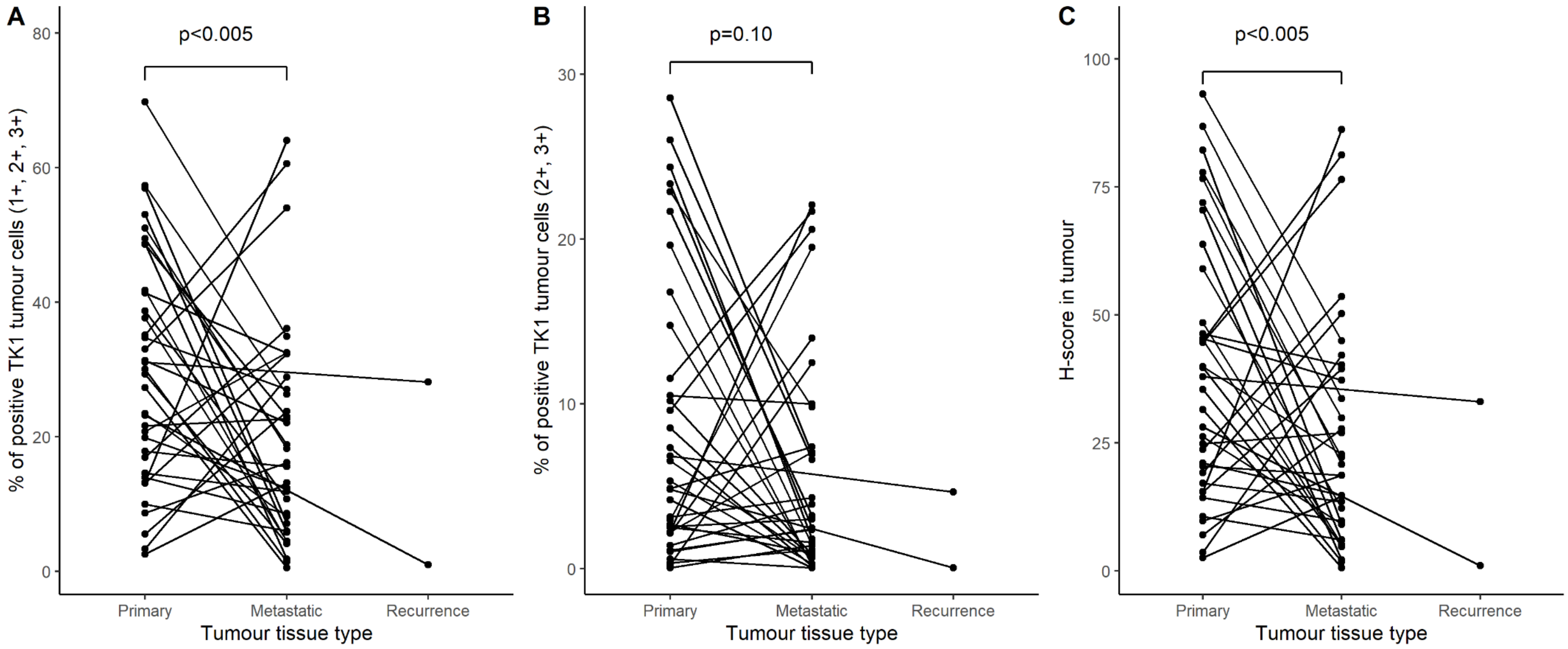

Paired scatterplots indicating the TK1 expression level in patients for which paired tissue samples were available (primary tumour, metastatic or recurrence tissue samples). The percentage of TK1 expressing tumour cells (+1,+2, +3), percentage of TK1 expressing tumour cells (+2, +3) and H-score are represented in the y-axis for plots A, B and C, respectively. The paired samples t-test *p*-value is indicated in the plot for the primary and metastatic tissue samples.

Supplementary Figure S6. TK1 expression levels per tissue type

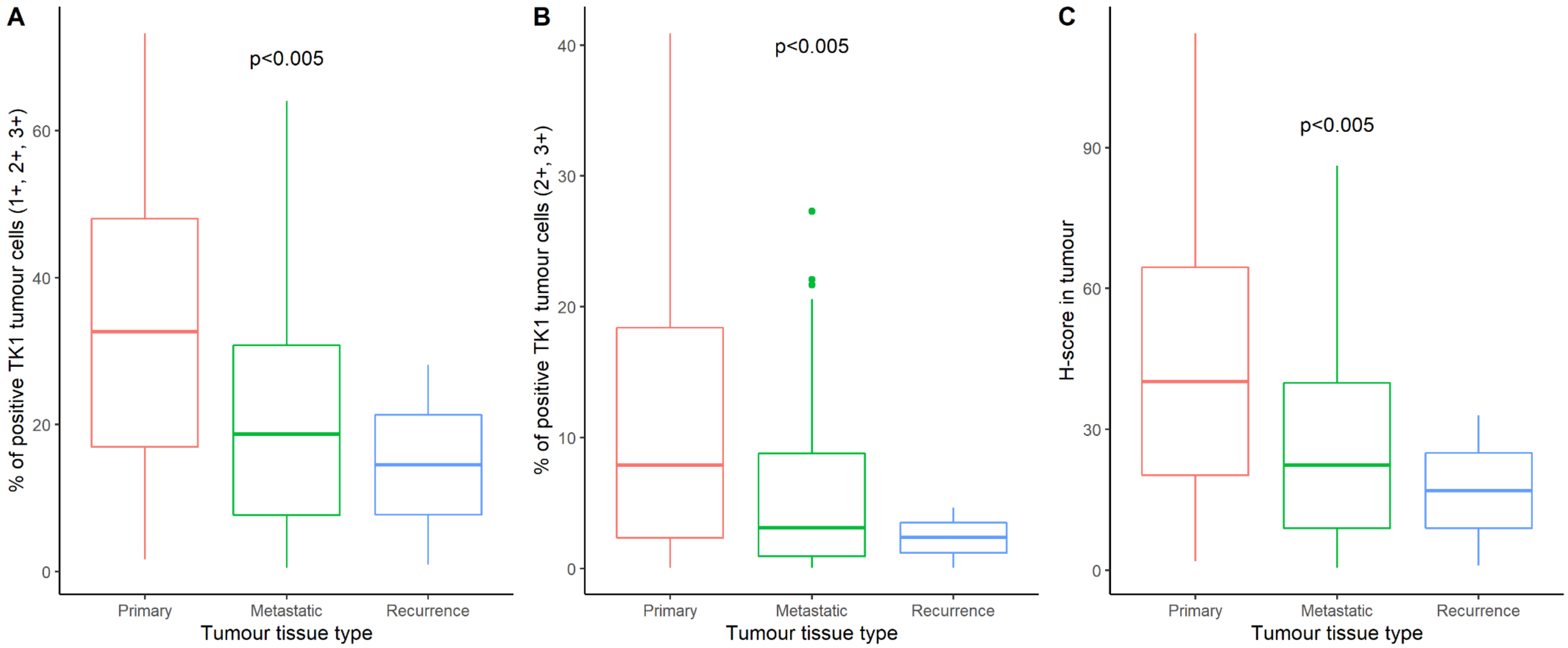

Boxplots indicating the TK1 expression level for all patients for which tissue samples were available, categorized per type of tissue sample (primary tumour, metastatic or recurrence tissue samples). The *p*-values for the Kruskal-Wallis test are shown. The percentage of TK1 expressing tumour cells (+1,+2, +3), percentage of TK1 expressing tumour cells (+2, +3) and H-score are represented in the y-axis for plots A, B and C, respectively.

Supplementary Table S2. Progression-free survival in patients with low versus high TK1 expression levels based on different cut-off values

		All tissue samples			Primary tumour			Metastatic tissue		
		n	**PFS (median in days; 95% C.I.)**	***p*-value**	***n***	**PFS (median in days; 95% C.I.)**	***p*-value**	***n***	**PFS (median in days; 95% C.I.)**	***p*-value**
% TK1 expressing tumour cells (+2,+3)	< 2.5%	*51*	104 (86-158)	0.032	*25*	133 (89-238)	0.021	*25*	97 (67-186)	0.718
	≥ 2.5%	*100*	84 (72-98)	0.032	*72*	84 (72-98)	0.021	*27*	90 (70-180)	0.718
% TK1 expressing tumour cells (+2,+3)	< 5%	*69*	98 (87-136)	0.071	*36*	98 (89-192)	0.113	*31*	104 (79-211)	0.413
	≥ 5%	*82*	83 (71-98)	0.071	*61*	83 (71-108)	0.113	*21*	84 (65-180)	0.413
% TK1 expressing tumour cells (+2,+3)	< 10%	*97*	97 (86-123)	0.104	*55*	98 (86-141)	0.300	*41*	96 (84-141)	0.209
	≥ 10%	*54*	78 (68-104)	0.104	*42*	81 (68-112)	0.300	*11*	70 (65-NR)	0.209
% TK1 expressing tumour cells (+2,+3)	< 15%	*113*	97 (85-115)	0.245	*66*	93 (79-123)	0.815	*45*	97 (84-180)	0.017
	≥ 15%	*38*	81 (64-112)	0.245	*31*	84 (64-163)	0.815	*7*	65 (51-NR)	0.017
% TK1 expressing tumour cells (+2,+3)	< 20%	*127*	97 (85-112)	0.406	*77*	97 (79-115)	0.850	*48*	96.5 (84-141)	0.090
	≥ 20%	*24*	81 (64-115)	0.406	*20*	83.5 (64-180)	0.850	*4*	61 (51-NR)	0.090
% TK1 expressing tumour cells (+2,+3)	< 30%	*146*	92 (84-108)	0.001	*91*	92 (84-114)	0.097			
	≥ 30%	*5*	55 (50-NR)	0.001	*6*	56 (50-NR)	0.097			

Log-rank test *p*-values are shown. *Abbreviations:* C.I. (confidence interval), *n* (count), PFS (progression-free survival).

Supplementary Table S3. Overall survival in patients with low versus high TK1 expression levels based on different cut-off values

			**All tissue samples**			**Primary tumour**			**Metastatic tissue**	
		n	**OS (median in days; 95% C.I.)**	***p*-value**	***n***	**OS (median in days; 95% C.I.)**	***p*-value**	***n***	**OS (median in days; 95% C.I.)**	***p*-value**
% TK1 expressing tumour cells (+2,+3)	< 2.5%	*51*	273 (214-416)	0.385	*25*	298 (210-503)	0.209	*25*	242 (166-425)	0.857
	≥ 2.5%	*100*	234 (176-266)	0.385	*72*	239 (191-278)	0.209	*27*	210 (164-424)	0.857
% TK1 expressing tumour cells (+2,+3)	< 5%	*69*	246 (210-400)	0.679	*36*	246 (199-425)	0.565	*31*	273 (210-422)	0.682
	≥ 5%	*82*	239 (171-278)	0.679	*61*	244 (191-293)	0.565	*21*	167 (155-452)	0.682
% TK1 expressing tumour cells (+2,+3)	< 10%	*97*	239 (199-281)	0.834	*55*	246 (199-298)	0.709	*40*	234 (166-416)	0.613
	≥ 10%	*54*	242 (171-408)	0.834	*42*	242 (171-408)	0.709	*12*	219 (164-NR)	0.613
% TK1 expressing tumour cells (+2,+3)	< 15%	*113*	242 (199-281)	0.672	*66*	244 (199-298)	0.397	*45*	239 (167-416)	0.343
	≥ 15%	*38*	244 (164-438)	0.672	*31*	249 (167-439)	0.397	*7*	164 (91-NR)	0.343
% TK1 expressing tumour cells (+2,+3)	< 20%	*127*	242 (199-281)	0.417	*77*	246 (199-295)	0.294	*48*	234 (167-408)	0.559
	≥ 20%	*24*	244 (167-452)	0.417	*20*	244 (167-NR)	0.294	*4*	209 (91-NR)	0.559
% TK1 expressing tumour cells (+2,+3)	< 30%	*146*	242 (211-281)	0.335	*92*	249 (211-295)	0.300			
	≥ 30%	*5*	111 (71-NR)	0.335	*5*	111 (71-NR)	0.300			

Log-rank test *p*-values are shown. *Abbreviations:* C.I. (confidence interval), *n* (count), OS (overall survival).
